# On the Analysis of Cryogels and Xerogels Using Cellulose Nanofibers and Graphene Oxide

**DOI:** 10.3390/polym15183833

**Published:** 2023-09-20

**Authors:** Bianca Cristina Moggio, Rosangela Bergamasco, Cid Marcos Gonçalves Andrade, Linnyer Beatrys Ruiz Aylon

**Affiliations:** 1 Department of Chemical Engineering, State University of Maringá, Maringá 87020-900, Brazil; pg403463@uem.br (B.C.M.); rbergamasco@uem.br (R.B.); 2 Department of Informatics, State University of Maringá, Maringá 87020-900, Brazil; lbruiz@uem.br

**Keywords:** nanomaterials, aerogel, graphene, cellulose, hydrothermal reduction

## Abstract

Aerogels are highly porous and ultralight three-dimensional materials with great potential for various applications. To obtain highly porous and structurally stable aerogels, a carefully designed synthesis process is required. These materials offer flexibility in manipulating their properties, allowing the incorporation of modifying agents according to specific needs. In this study, compounds were synthesized using graphene oxide (GO) and nanocellulose fibers (NFC) through the hydrothermal reduction methodology. Two drying techniques were employed: lyophilization and oven evaporation, resulting in materials called cryogel and xerogel, respectively. Various parameters that can interfere with the properties of these nanomaterials were evaluated. The results indicated that the cryogel dried by lyophilization provided the best applicability due to its structural flexibility after compressions, whereas the xerogel obtained through the oven evaporation process resulted in a compound with high rigidity and disintegration. Structural characterizations demonstrated the successful development of the precursors and promising characteristics in the synthesized nanomaterials. With its flexibility, approximately 98% porosity, low shrinkage rate, light weight, and electrical conductivity, the developed cryogel showed high potential in various applications, such as pressure sensors, electromagnetic shielding, and other research and development fields.

## 1. Introduction

Aerogels are three-dimensional materials with an adjustable porous structure and low density. This class of materials offers significant potential in terms of scalability and allows the manipulation of properties to meet different needs [1]. They are obtained by replacing the liquid present in the material with gas, resulting in a highly porous structure with a three-dimensional network of interconnected empty spaces [2].

The synthesis of these materials involves steps such as gelation, aging, and drying. In gelation, electrostatic interactions occur between nanoparticles and the solvent, known as sol–gel chemistry. Aging contributes to the formation of a stable three-dimensional structure. Drying is the most critical stage of the process, where the solvent is gradually removed to preserve the porous structure of these materials [3,4].

In the sol–gel formation process, aerogels allow the synthesis of modifying agents that promote the chemical bonds and mechanical stability of the compound [5,6]. These materials can be derived from organic, inorganic, or even hybrid precursors, which opens the way for a wide range of applications in various fields [7,8].

Due to the high amount of oxygen-containing functional groups, such as carboxyl, hydroxyl, and epoxide groups, GO exhibits high reactivity for covalent functionalization [9,10]. Synthesized through methodologies involving the use of oxidizing agents and strong acids, its intrinsic properties, such as hydrophilicity, high surface area, and good thermal properties, make it a promising compound to be synthesized into aerogels [11,12].

Although the properties are attractive, these porous GO materials exhibit vulnerable mechanical performance when subjected to compressions due to the oxidation conditions and weak Van der Waals interactions between nanosheets, as well as the weakening of π–π stacking interactions [13,14]. In this context, it is necessary to seek natural structural reinforcements from renewable and widely available sources in nature as potential candidates to replace synthetic polymers [15].

Cellulose (CE), obtained from plants and agricultural residues, is a widely available renewable resource. Composed of glucose units connected by β(1,4)-glycosidic linkages, CE has an abundance of hydroxyl groups, allowing for the formation of inter- and intramolecular hydrogen bonds. These characteristics provide stability and a stable structure when synthesized into polymeric matrices [16,17].

NFC have physicochemical properties that can be used as stabilizers or dispersants for 2D materials [18,19]. The process of isolating these fibers on a nanometer scale results in a structure with higher mechanical stability, due to a more uniform distribution of particle size [20,21,22]. Additionally, NFC also contributes to a better distribution of GO sheets during the hydrothermal treatment, significantly reducing the formation of agglomerated sheets and allowing for greater porosity [23].

The aging step leads to the formation of three-dimensional networks, using hydrothermal reduction through pressurized systems and controlled temperatures. This method promotes phase separation by dehydrating the GO. This methodology results in structured materials, eliminating the need for additional treatments. However, strict control of this process is essential to ensure the proper balance between porosity and stable structure [24,25].

The three-dimensional networks with high porosity can be preserved almost unchanged through well-developed aging and drying techniques. Depending on the drying strategy used, these materials receive different denominations. Cryogels are produced under cryogenic conditions, that is, by freezing the samples and thawing them using freeze-drying [26]. On the other hand, xerogels are materials developed through drying by evaporation in low-temperature ovens [4].

A combination of GO and NFC in these nanomaterials can lead to improved properties compared to materials developed with pure GO. The presence of NFC also enhances the distribution of GO, increasing structural stability and strengthening the resulting mechanical properties. This opens possibilities for applications in areas such as sensing [27,28,29], supercapacitors [30,31,32], pollutant adsorption processes in water [33,34,35], flame retardants [36], electromagnetic interference shielding [37,38,39], and solar-to-thermal energy conversion [40,41], among other fields of research and development.

For this purpose, this study investigated different routes for the synthesis through the hydrothermal reduction route. The aim was to evaluate the behavior of the synthesized GO and NFC precursors, aiming to obtain highly porous and flexible nanomaterials. Different drying techniques were evaluated, such as freeze-drying and controlled-temperature oven evaporation, resulting in the production of materials called cellulose nanofiber-synthesized graphene oxide cryogels (CGO-NFC) and cellulose nanofiber-synthesized graphene oxide xerogels (XGO-NFC), respectively. The main objective of this study is to guide researchers interested in developing highly porous and structurally stable three-dimensional nanomaterials through the synthesis of GO and NFC precursors using hydrothermal reduction. These composite materials can improve performance in various technological areas, such as sensing, solar–thermal–electric energy conversion, and electromagnetic interference shielding, providing innovative solutions for current challenges.

## 2. Materials and Methods

### 2.1. Synthesis of GO

GO films were developed following the methodology proposed by Hummers Jr and Offeman [11], with the addition of a graphite pre-oxidation step. This methodology involves the chemical exfoliation of graphite using strong acids to achieve the separation of layers and obtain a single oxidized layer, called GO. Initially, 5 g of graphite was pre-oxidized in a reflux system containing 2.5 g of phosphorus pentoxide (P${}_{2}$O${}_{5}$), 2.5 g of potassium persulfate (K${}_{2}$S${}_{2}$O${}_{8}$), and 18 mL of sulfuric acid (H${}_{2}$SO${}_{4}$). The mixture was heated to 80 °C and stirred for 5 h. After centrifugation and drying in an oven at 60 °C for 12 h, 1 g of the pre-oxidized material was stirred with 23 mL of H${}_{2}$S$O_{4}$, and then 3 g of potassium permanganate $KMnO_{4}$ was added slowly and in small doses to control the temperature.

After 2 h of stirring at 35 °C, 46 mL of deionized water was added, and the reaction was stirred for an additional 2 h. Subsequently, 140 mL of water and 2.5 mL of 30% hydrogen peroxide (H${}_{2}$O${}_{2}$) were added, and to conclude the process, 250 mL of 10% hydrochloric acid (HCl) aqueous solution was added to the mixture, which was then left at rest for 24 h. The final step involves several washes to remove the supernatant and any undesired components, achieving a constant pH. The mixture was then centrifuged and uniformly transferred to a Petri dish for drying at 60 °C for 12 h to obtain the GO films.

### 2.2. Synthesis of NFC

The extraction of NFC was performed through the delignification and bleaching of fibers obtained from the discarded green coconut mesocarp. The fibers in their natural state underwent a pre-treatment with hot water to remove soluble agents. Then, they were dried in an oven, crushed, and sieved through a 100 mesh sieve.

Next, for delignification, 2% sodium hydroxide (NaOH) was used, maintaining constant stirring at a temperature of 80 °C for 3 h. The main objective of this alkaline step was to break the covalent interactions between lignocellulose and hemicellulose. The resulting pulp was subjected to vacuum filtration and washed with distilled water to ensure the complete removal of soluble agents.

Following that, the bleaching process was carried out using a mixture of 30% hydrogen peroxide (H${}_{2}$O${}_{2}$) and 2% sodium hydroxide (NaOH) in a 1:1 ratio. This process occurred under agitation at temperatures between 60–80 °C for 3 h, followed by additional washes until a neutral pH was achieved.

### 2.3. Synthesis of CGO-NFC and XGO-NFC

**Figure 1:**
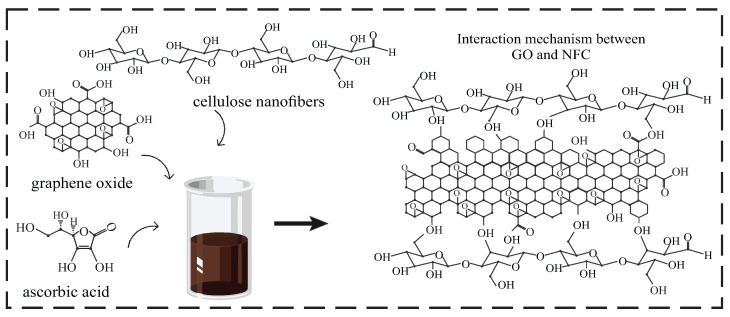
Interaction mechanism between GO and NFC.

The synthesis of CGO-NFC and XGO-NFC nanomaterials was carried out using a modified hydrothermal treatment process. In summary, GO suspensions containing the reducing agent ascorbic acid (AA) were subjected to an ultrasound bath for complete homogenization. Then, the NFC mass was added and maintained under ultrasound. Subsequently, the solution was transferred to a Teflon-coated autoclave and maintained at a certain temperature and time, showing interactions between the GO and NFC precursors, as shown in Figure 1. After investigating the pH of the solution, reduction steps, time, and temperature, the nanomaterials underwent different drying techniques: lyophilization and evaporation in an oven, resulting in products called cryogel and xerogel, respectively.

### 2.4. Influence Factors on the Developed Nanomaterials

To investigate the factors affecting nanomaterial formation, such as the dosage of the reducing agent AA, the GO:NFC ratio, pH, hydrothermal reduction steps, time, and temperature, systematic analyses were conducted using the one-factor-at-a-time method. The results demonstrated that these parameters have a significant impact on the development of the nanomaterials.

Initially, the GO concentration was fixed at 5 mg/mL, keeping the solvent volume constant at 50 mL of deionized water, whereas the mass dosage of the reducing agent AA was varied at GO:AA ratios of 1:1, 1:2, and 1:3. Next, different ratios of GO:NFC (1:0.25, 1:0.5, 1:1, 1:2, 1:3, and 1:4) were evaluated, maintaining the initial mass concentration of GO relative to the solvent. The weight of this precursor was measured after the vacuum filtration of its aqueous suspension.

Additionally, experiments were conducted to evaluate the effect of the pH of the nanoparticle mixture and the solvent, using acidic solutions (pH ≈ 3), neutral solutions (pH ≈ 7), and basic solutions (pH ≈ 10), while keeping the optimal experimental conditions for AA and NFC unchanged.

In order to achieve an appropriate balance between mechanical structure and porosity, comparative analyses were performed on nanomaterials subjected to one, two, and three stages of hydrothermal reduction. It was also essential to study the precise control of temperature, varying at 80 °C, 100 °C, and 150 °C, as well as the reduction time, ranging from 3, 6, 12, and 24 h.

These investigations have provided valuable insights for the development of synthesis methods for GO:NFC nanomaterials. Understanding the influences of synthesis parameters, such as the dosage of the reducing agent, the proportion of components, and other relevant factors, enables precise control and adjustment of the characteristics. This way, it becomes possible to obtain materials with suitable mechanical properties, such as strength and flexibility, while maintaining high porosity, allowing for a wide range of applications.

### 2.5. Characterizations

The characterization techniques employed to assess the morphology of the precursors and nanomaterials included scanning electron microscopy (SEM) using a Shimadzu SS-550 and Tescan Vega 4 model, as well as transmission electron microscopy (TEM) utilizing a Jeol JEM-1400Flash Model. These techniques were utilized to analyze the chemical groups present in the samples. Furthermore, the density, shrinkage, and porosity of CGO-NFC and XGO-NFC were investigated. The porosity (*P*) of the samples was determined using Equations (1)–(3):

(1) $$P(\%)=(1-\left(\rho_{a}/\rho_{s}\right))\times 100$$

(2) $$\rho a=\frac{m}{V}$$

(3) $$\rho s=(1/\left(\frac{W_{GO}}{\rho_{GO}}+\frac{W_{NFC}}{\rho_{NFC}}\right))$$

where $\rho_{a}$ represents the apparent density and $\rho_{s}$ represents the density of the precursors. Moreover, *m* is the weight of the samples in grams, and the volume (*V*) was calculated by measuring the diameter (*d*) and height (*h*) of the cylindrical samples in cubic meters (m${}^{3}$). $W_{GO}$ represents the mass fraction of GO in the sample, and $W_{NFC}$ is the mass fraction of NFC. The densities used were found in the literature, with $\rho_{GO}$ and $\rho_{NFC}$ being 1.06 g/cm${}^{3}$ and 1.5 g/cm${}^{3}$, respectively [42,43,44]. To calculate the shrinkage of these materials, the initial and final volumes were analyzed after the drying techniques.

For structural analysis, Fourier-transform infrared spectroscopy (FTIR) was used with the Bruker Vertex 70v FTIR model in a spectral range of 4000 to 400 cm${}^{-1}$. Additionally, Micro-Raman spectroscopy was performed using the Senterra model, with an excitation wavelength of 532 nm and a spectral range of 4000 to 400 cm${}^{-1}$, with a spectral resolution of 9 to 15 cm${}^{-1}$.

An X-ray diffraction (XRD) technique was also used with the Bruker D8 Advance model, equipped with a Cu radiation source (wavelength λ = 1.5406 Å), operating at 40 kV and 30 mA. The scan was performed in the range of 5° to 100°, with a scanning speed of 0.5° per minute. Additionally, the Bragg equation, as presented in Equation (4), was employed to estimate the interlayer distance [45]:

(4) λ=2×d×senθ

where λ represents the wavelength of the copper (Cu) X-ray beam, *d* is the spacing between crystal layers, and θ is the diffraction angle. X-ray diffraction also allows the determination of the crystallinity index (*CI*) of the CE samples, which is relevant information extracted from the XRD spectra and developed by Segal et al. [46]. The method involves the ratio between the height of the crystalline peak intensity ($I_{002}$) and the intensity of the amorphous peak ($I_{AM}$), as given by Equation (5):

(5) $$CI=\frac{I_{002}-I_{AM}}{I_{002}}\times 100$$

Stress–strain curves were also conducted on a universal materials testing machine, model LLOID LR10KPlus, and the thermal behavior of the nanomaterials was analyzed using thermogravimetric analysis (TGA) with the Shimadzu TGA-50 model, over a temperature range of 25 to 800 °C. This provided a comprehensive understanding of the thermal behavior of the CGO-NFC and XGO-NFC compounds.

## 3. Results and Discussion

### 3.1. Factors Defined for the Nanomaterials

After a careful analysis of the aforementioned parameters, it was possible to identify the optimal conditions for the development of the nanomaterials in this study, as presented in Table 1. The optimal ratio of the reducing agent to GO was determined to be 1:3 (GO:AA). The results revealed that the reduction of the material showed variation in porosity as the AA proportion increased. This behavior can be attributed to the modification of the hybrid $sp^{3}$-to-$sp^{2}$ carbon structure by the reducing agent, resulting in an increase in π–π stacking forces in the nanomaterial.

**Figure 2:**
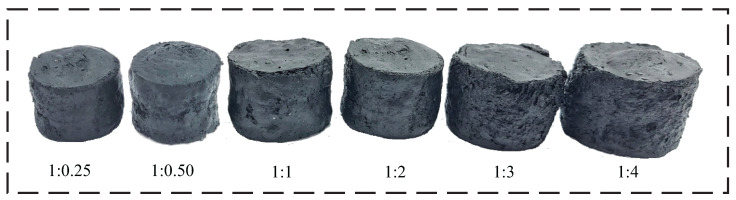
Images of nanomaterials before the drying process with varying GO:NFC ratios.

Regarding the GO:NFC precursor ratio, the results obtained after hydrothermal reduction without the application of the drying process, as shown in Figure 2, demonstrated an increase in dimensions and porosity as the NFC proportion increased. Additionally, it was observed that the lower ratio of 1:0.25 yielded the best results for CGO-NFC after the lyophilization drying process. On the other hand, for XGO-NFC, the ratio did not play a significant role, as both the lower and higher ratios resulted in reduced flexibility after evaporation drying.

Additionally, the mixture solution was adjusted to an acidic pH for both compounds. It was observed that when the pH was adjusted to 7 and 10, the material’s structure became fragile, leading to the disintegration of the skeleton and the material exhibiting low mechanical properties.

**Figure 3:**
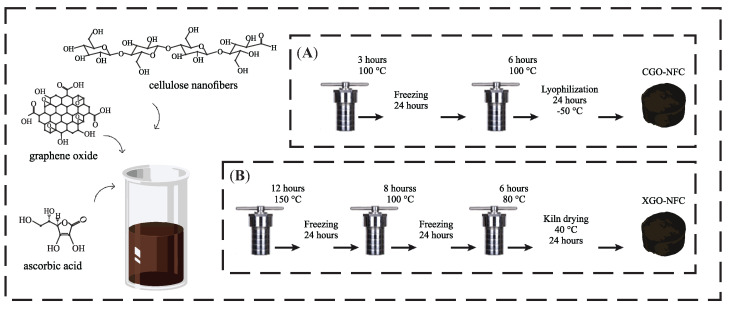
Schematic and summary illustration of the synthesis methodology for the development of (**A**) CGO-NFC and (**B**) XGO-NFC.

The synthesis temperature and reduction steps for CGO-NFC, as shown in Figure 3, were carried out at 100 °C and divided into two steps, with the first step lasting 3 h and the second step being 6 h of reduction. For XGO-NFC, the reduction was conducted in three distinct steps, with the first step lasting 12 h at a temperature of 150 °C, followed by an 8 h step at 100 °C, and a third step of 6 h at 80 °C. In both cases, the compounds were subjected to freezing for 24 h between the hydrothermal reduction steps.

It was found that after synthesizing the nanomaterials at 80 °C with just one hydrothermal reduction step, regardless of the temperature, it was necessary for the material to undergo another hydrothermal reduction step due to the low degree of structuring at this temperature, as well as the weakness in π–π interactions between the layers of GO.

On the other hand, when synthesizing the nanomaterials at 150 °C, the need for a careful study of the reduction steps was observed, as intense reduction stimulates the precipitation of GO. This implies that a careful control of the reduction steps is crucial to obtain GO nanomaterials with the desired characteristics.

The CGO-NFC was subjected to freeze-drying at a temperature of −50 °C for 24 h, thus preserving the flexibility of the compound. On the other hand, the XGO-NFC was dried by evaporation in a controlled oven at 40 °C for 24 h; however, this drying technique did not contribute to the flexibility of the compound. To achieve the desired result, it was necessary to use the lowest proportion of the NFC precursor. This is because, as the NFC proportion increases, the compound tends to become more rigid.

After conducting the tests, it was found that in the case of CGO-NFC, two reduction steps with relatively short times were sufficient to obtain a nanomaterial with the desired characteristics, whereas a single step resulted in a fragile nanomaterial. For XGO-NFC, it was observed that the reduction steps should be performed gradually, as when subjected to oven-drying with a lower number of steps and lower reduction temperatures, the collapse of porosity occurred, resulting in an excessively rigid material.

### 3.2. Morphological Characterization

**Figure 4:**
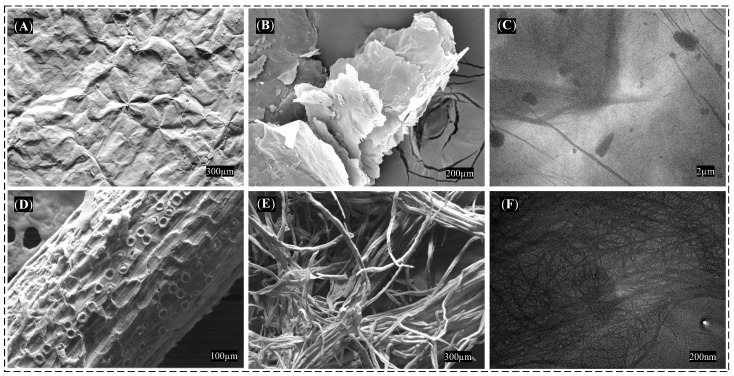
Morphological images: (**A**,**B**) surface and stacked layers of GO sheets by SEM, (**C**) superimposed layers of GO sheets by TEM, (**D**,**E**) raw fiber and fiber post-treatment by SEM, (**F**) NFC by TEM.

The characterizations conducted via SEM and TEM in Figure 4 aimed to investigate the morphology of the GO and NFC precursors. In Figure 4A, the typical surface morphology of GO can be observed, featuring wrinkles. Figure 4B illustrates the layer stacking in the GO sheets, whereas Figure 4C presents the overlapping layers as observed by TEM.

Figure 4D portrays the morphology of the natural coconut fiber with impurities; Figure 4E showcases the morphology of treated nanofibers, indicating the removal of hemicellulose and lignin after chemical treatment; and Figure 4F displays the post-processed NFC at the nanoscale, as observed through TEM.

**Figure 5:**
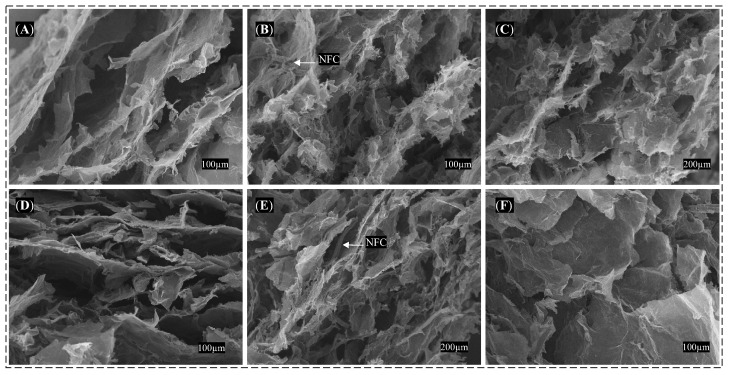
Morphological images obtained by SEM: (**A**–**C**) CGO-NFC and (**D**–**F**) XGO-NFC.

Figure 5A–C depict the morphology obtained by SEM of the CGO-NFC, whereas Figure 5D–F present the XGO-NFC. These compounds showcase a three-dimensional structure composed of GO sheets supported by NFC, which are interconnected and form a porous network.

This porous architecture is accountable for the material’s low weight, rendering it ultralight in performance. Moreover, the presence of NFC in both compounds, as evidenced in the figures between the GO layers, also enhances the mechanical properties of the composites, imparting them with greater malleability.

Another analysis performed on the nanomaterials was the density, shrinkage, and porosity in triplicate using Equations (1)–(3) to ensure the accuracy of the results, as shown in Table 2. They exhibited remarkably low density, around 0.0154 and 0.0249 g/cm${}^{3}$, respectively, due to the porous structure composed of a three-dimensional network of interconnected pores for CGO-NFC and XGO-NFC.

Furthermore, CGO-NFC exhibited a lower shrinkage compared to XGO-NFC, with an average of 2.86% and 6.54%, respectively. This notable difference is due to the drying technique employed. The low shrinkage rate of CGO-NFC indicates a more stable structure compared to XGO-NFC. Both materials showed high porosity, reaching an average of 98.62% and 98.19%. According to Aegerter et al. [47], porosity values above 85% and shrinkage below 35% are characteristics that classify materials as aerogels.

### 3.3. Structural Characterization

**Figure 6:**
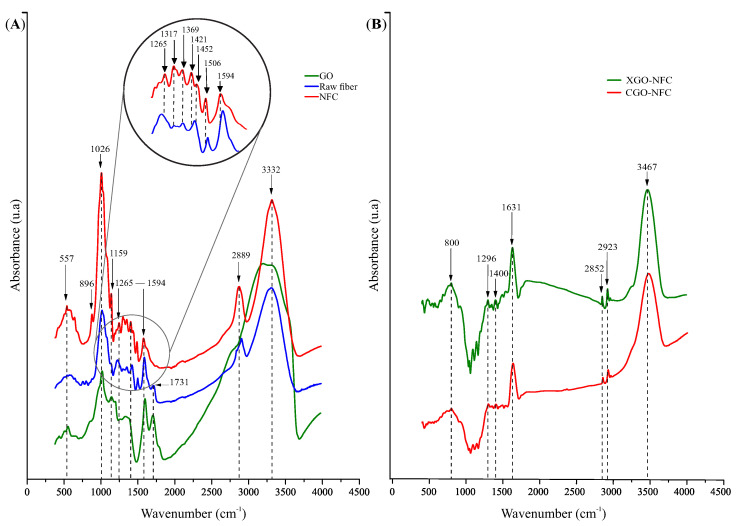
FTIR spectra of (**A**) GO, raw fiber, and NFC; and (**B**) CGO-NFC and XGO-NFC.

FTIR is a technique that provides spectra for the identification and characterization of molecules based on the frequency regions of functional groups. The correlation between characteristic vibrations and functional groups is summarized in Table 3, considering references in the literature [33,34,48,49,50,51,52,53]. Figure 6A shows the main spectra for the identification of GO, raw fiber, and NFC samples, whereas Figure 6B presents the spectra obtained for the CGO-NFC and XGO-NFC nanomaterials.

The characteristic vibrations and functional groups of GO are observed at the peak of 578 cm${}^{-1}$ with methyl group bending vibrations (C-H). The peak at 875 cm${}^{-1}$ is associated with the aromatic ring deformation vibration in C-H, and the peak at 1039 cm${}^{-1}$ corresponds to the identification of primary alcohol in the structure. Another stretching vibration peak at 1164 cm${}^{-1}$ is associated with the C-O stretching vibration of the epoxy group, due to the incorporation of oxide functional groups.

Similarly, the vibration at 1218 cm${}^{-1}$ is related to the carboxylic acid group. The peak observed at 1620 cm${}^{-1}$ is due to the effect of C=C skeletal vibrations in $sp^{2}$ domains. The stretching vibrations at 1726 cm${}^{-1}$ are associated with the carboxylic acid group, and the broad band observed between 3213 and 3319 cm${}^{-1}$ is attributed to the stretching vibrations of hydroxyl groups and absorbed water molecules.

For the natural fibers and NFC, it is possible to observe the peak at 896 cm${}^{-1}$ with stretching vibrations of the C-H bond, characterized by the deformation of hemicellulose through the curve of the aromatic ring, but it can also be identified in the amorphous region of cellulose. Prior to this, peaks of stretching vibrations of C-H bonds are characterized, but in this case, they are attributed to the deformation of lignin; however, the peaks of the natural fibers presented in the graph are smooth.

The peak with stretching vibration found at 1026 cm${}^{-1}$ in NFC is also identified in natural fibers, but with a lower intensity at 1035 cm${}^{-1}$, resulting from C-O bonds in symmetric stretching. The most significant changes between the peaks of NFC and natural fibers are presented between the peaks at 1159 cm${}^{-1}$ to 1506 cm${}^{-1}$ in NFC, whereas in natural fibers, the peaks in the spectrum are drastically reduced between 1155 cm${}^{-1}$ to 1517 cm${}^{-1}$.

The vibration peak at 1608 cm${}^{-1}$ in natural fibers was reduced in NFC with a peak at 1594 cm${}^{-1}$. It is also observed that the peak at 1731 cm${}^{-1}$ present in natural fibers is not found in NFC, due to the effective reduction of lignin and hemicellulose. The peak at 2889 cm${}^{-1}$ is attributed to the stretching vibration of C-H hydrocarbons in the polysaccharide constituents, which are generally compatible with the presence of carbon with $sp^{3}$ hybridization. The characteristic stretching peak at 3332 cm${}^{-1}$ is identified by the O-H hydroxyl group in polysaccharides and also includes inter- and intramolecular hydrogen bonding vibrations in NFC.

In the nanomaterials, the vibration peaks at 796–800 cm${}^{-1}$ are characterized by the deformation of C-H bonds in the aromatic rings of GO. The vibration peak at 1298–1296 cm${}^{-1}$ is related to the presence of functional groups containing C-O bonds in the nanomaterials. This interaction can be attributed to specific chemical bonds or intermolecular forces between the GO and NFC precursors [52].

The peak at 1402–1400 cm${}^{-1}$ indicates an asymmetric deformation vibration of $CH_{3}$ groups. The peak around 1631 cm${}^{-1}$ corresponds to the absorption peak of C=C stretching vibration, indicating the presence of these bonds in the material. The peaks at 2852 and 2921–2923 cm${}^{-1}$ correspond to the stretching vibrations of methyl groups, whereas the vibration peak at 3467 cm${}^{-1}$ is related to the hydroxyl (OH) group.

**Figure 7:**
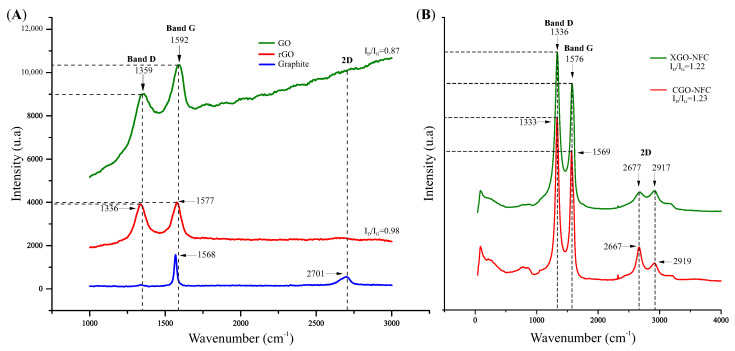
Raman spectra of (**A**) GO, rGO, and graphite; and (**B**) CGO-NFC and XGO-NFC.

The Raman spectra shown in Figure 7A provide the peaks of commercial graphite, GO, and rGO, and Figure 7B shows the spectra of the nanomaterials CGO-NFC and XGO-NFC. Three main features of carbonaceous materials and their derivatives are found in the Raman spectra: the D band, G band, and 2D band [54]. The D band is generally characterized by structural imperfections resulting from oxygenated groups after oxidation reactions present in the carbon. The intensity of the D band decreases with an increase in the material’s crystallinity, consequently reducing the number of defects [55].

The G band is related to the formation of the hybridized $sp^{2}$ carbon network, representing the quality of the graphitic network of the material. In other words, the higher the peak intensity in relation to the D band, the lower the $I_{D}/I_{G}$ ratio, indicating fewer structural defects in the material. On the other hand, the second-order of the D band, known as the 2D band, is related to the number of layers in the material. Thus, the spectra exhibit higher intensity, as the material has a greater number of GO layers stacked together. This is evident in the peak at 2701 cm${}^{-1}$ in the graphite spectrum, whereas the GO and rGO spectra do not show significant peaks.

In the GO and rGO spectra, it is evident that the D band peaks have lower intensity at 1359 cm${}^{-1}$ and 1336 cm${}^{-1}$, respectively, and the G band peaks have higher intensity at 1592 cm${}^{-1}$ for GO and 1577 cm${}^{-1}$ for rGO. The intensity ratio of the D and G bands ($I_{D}/I_{G}$) is a qualitative formula to assess the level of disorder in the developed material. When the $I_{D}/I_{G}$ ratio > 1, it indicates that the material has structural defects due to disruption in the $sp_{2}$ hybridization. On the other hand, when the $I_{D}/I_{G}$ ratio < 1, it characterizes a material with fewer structural defects and a better graphitic structural network, as presented with $I_{D}/I_{G}$ = 0.87 and 0.98 for the GO and rGO compounds, respectively.

The analysis of the Raman spectra of the CGO-NFC and XGO-NFC compounds, presented in Figure 7B, allowed for the evaluation of the quality of the materials developed by lyophilization and controlled evaporation, respectively. The presence of disorder and imperfections in the structural network is evidenced by the peaks at 1333 and 1336 cm${}^{-1}$, related to the D band. The G band, attributed to the vibrations of the GO network, indicates the presence of $sp^{2}$ hybridized carbon in the nanomaterials and is represented by the peaks at 1569 and 1576 cm${}^{-1}$.

The 2D band is also visible, with vibrations at 2667 and 2919 cm${}^{-1}$ in CGO-NFC and 2677 and 2917 cm${}^{-1}$ in XGO-NFC. This band is related to the number of stacked layers of GO present in the compound. It can be observed that the $I_{D}/I_{G}$ ratio for CGO-NFC is 1.23 and for XGO-NFC is 1.22, indicating the presence of structural defects, such as disorder and imperfections in the carbon network, in both compounds. These results indicate that the materials exhibited characteristics of structural defects.

**Figure 8:**
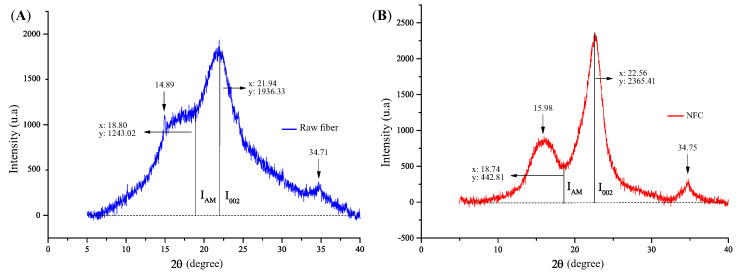
Crystallinity of (**A**) raw fiber and (**B**) NFC.

DRX patterns were performed on samples of raw coconut fibers and NFC. Raw coconut fibers exhibited three main peaks, as shown in Figure 8. After the delignification and bleaching treatment, which removed the lignin and hemicellulose fractions from the fibers, narrower and more intense crystalline peaks were observed for the modified fibers, along with a significant reduction in the amorphous content. The observed angles were 2θ = 15.98°, 22.56°, and 34.75°

To identify the crystallinity, Equation (5) was applied, and the proposed method was performed on the natural coconut fiber and NFC, calculating the ratio between the height of the peak ($I_{002}$) and the amorphous height ($I_{AM}$), resulting in 35.8% and 81.3%, respectively. This indicates an increase of 56% in crystallinity due to the successful removal of non-crystalline compounds such as lignin, hemicellulose, and pectin present in the natural coconut fiber.

**Figure 9:**
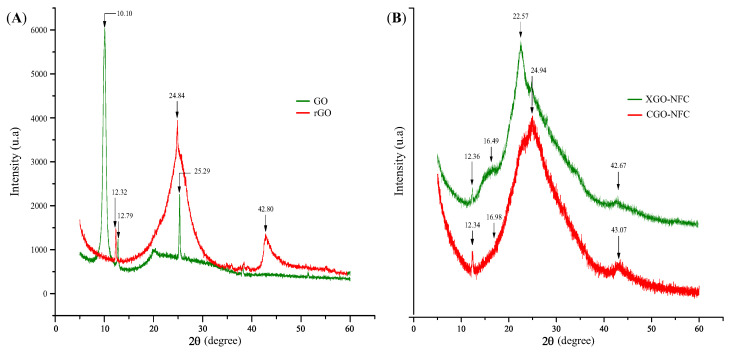
XRD diffraction of (**A**) GO and rGO precursors and (**B**) CGO-NFC and XGO-NFC nanomaterials.

To GO and rGO, as shown in Figure 9A, the Bragg equation was used to estimate the distance between the layers. The spectra after the oxidative reaction of graphite using the Hummers method showed functional groups dispersed in the basal plane, increasing the spacing between the layers, as demonstrated in the GO and rGO spectra. The GO spectrum exhibited a specific X-ray diffraction (XRD) pattern characterized by the main peak at a scattering angle of 2θ = 10.10° corresponding to the (001) plane and an interplanar distance of 0.88 nm, commonly reported in the literature [56].

For rGO, it can be observed that the main peak was presented at an angle of 2θ = 24.84° with a spacing of 0.36 nm corresponding to the (002) plane, slightly smaller than GO. This reduction in spacing may be attributed to the reductive removal of oxygen-containing functional groups trapped between the layers of GO. In contrast, the significant distance in GO is associated with the functionalization of graphite layers with oxygen-containing groups during the oxidation process.

The XRD patterns of the nanomaterials were obtained as shown in Figure 9B. In the spectra, it is possible to identify peaks that are consistent with the literature, corresponding to the crystalline planes (002) and (101). A decrease in the peaks at around approximately 12° compared to the GO XRD was observed, indicating its reduction. Furthermore, the characteristic peak of NFC at around approximately 16° is defined in the nanomaterials [57].

Furthermore, characteristic peaks of the synthesized materials with GO were observed in the (002) planes, with 2θ = 24.94° for CGO-NFC and 2θ = 22.57° for XGO-NFC, corresponding to spacings of d = 0.35 nm and d = 0.39 nm, respectively. Another characteristic peak of these compounds, widely reported in the literature, was also identified at 2θ = 43.07° for CGO-NFC and 2θ = 42.67° for XGO-NFC [29].

### 3.4. Thermal Characterization

**Figure 10:**
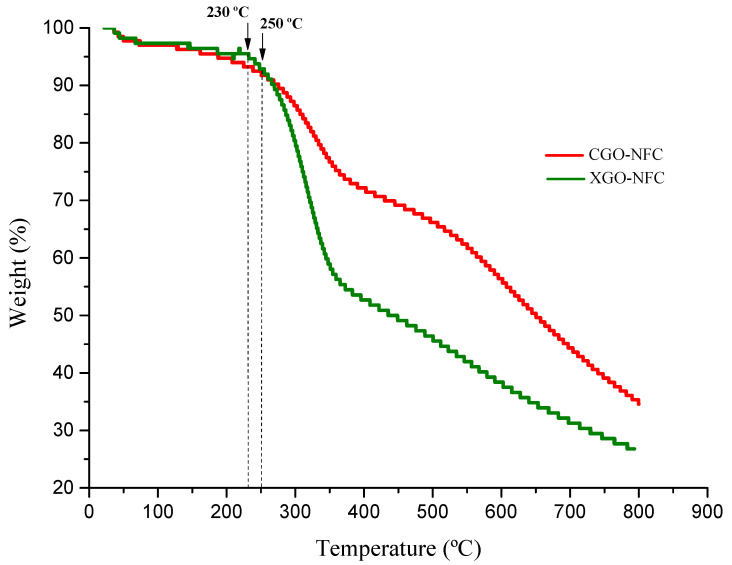
TGA curves of the compounds CGO-NFC and XGO-NFC.

Analysis TGA was performed on the nanomaterials to investigate their thermal behavior. Both materials showed a small weight loss from room temperature up to 100 °C, which may be attributed to the loss of water molecules present in the compounds. From this temperature, CGO-NFC exhibited a constant weight loss starting at 250 °C, whereas XGO-NFC began to show significant weight loss from 230 °C, as shown in Figure 10.

Possibly, the mass loss associated with the mentioned temperatures is related to the release of volatile species. Additionally, within this temperature range, the breaking of the glycosidic bonds present in the structure of NFC occurs in these materials.

### 3.5. Challenges and Important Issues

**Figure 11:**
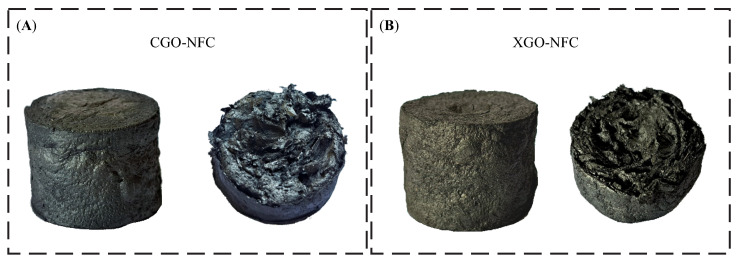
Images of the optimally developed nanomaterials (**A**) CGO-NFC and (**B**) XGO-NFC.

It was observed that both materials exhibited typical aerogel morphologies described in the literature, with low density, low shrinkage rate after the drying process, and high porosity, as shown in Figure 11. CGO-NFC showed slightly superior morphological characteristics in terms of lightness, shrinkage rate, and porosity compared to XGO-NFC. However, this difference is not significant enough to determine which material is better in this aspect.

The two compounds developed under the optimal conditions described in this study. It is possible to observe that both nanomaterials exhibit porosity in their cross-sections. However, there is a difference in the texture of their surfaces, with XGO-NFC being rougher compared to CGO-NFC. This indicates that the nanomaterials have distinct characteristics in terms of their surface morphology.

**Figure 12:**
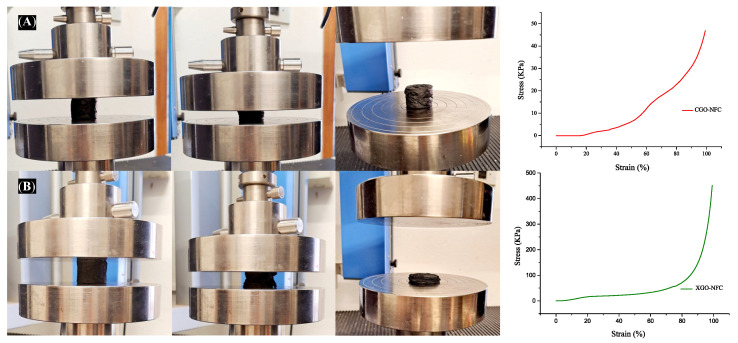
Images of the flexibility and stress–strain curves of the optimized developed nanomaterials, (**A**) CGO-NFC and (**B**) XGO-NFC, were obtained.

Compression analyses were also performed on the nanomaterials, which exhibited the characteristic behavior of porous materials. Figure 12A exemplifies the compression and decompression cycle of the CGO-NFC material, displaying a remarkable degree of malleability, as highlighted in the figure after decompression. The stress–strain curve began to exhibit a substantial increase in stress at deformations approaching 60% in the CGO-NFC nanomaterial.

Figure 12B illustrates the compression and decompression cycle of the XGO-NFC nanomaterial, depicting its rupture and collapse. Following compression, a notable lack of malleability was observed, as evidenced in the figure. The stress–strain curve revealed a substantial increase in stress at deformations approaching 80%.

**Figure 13:**
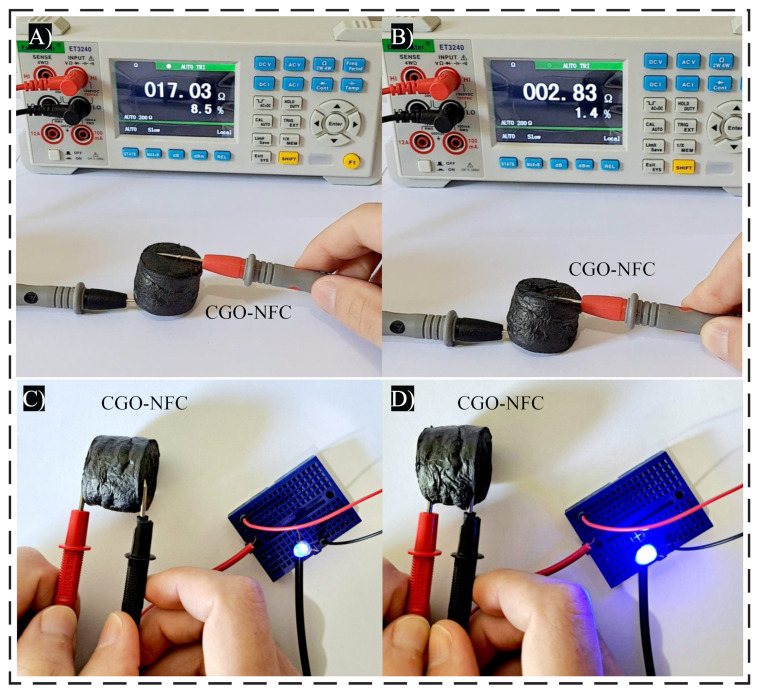
Resistance analysis of CGO-NFC nanomaterial using a bench multimeter (**A**) without compression and (**B**) with compression and conductivity analysis (**C**) without compression and (**D**) with compression.

These nanomaterials have a range of applications, one of which is their use in the field of pressure sensing, where a series of experiments can be conducted to investigate the electrical current response to applied pressure. As illustrated in Figure 13A,B, a decrease in electrical resistance is observed as the pressure is increased. Initially, in its unpressed state, only a few contact points occur within the CGO-NCE nanomaterial, forming limited conductive paths.

However, as pressure is applied, the distance between the inner walls of the pores decreases, and the number of conductive paths increases, resulting in a rapid reduction in the electrical resistance of the material. This behavior demonstrates the potential of these nanomaterials as pressure sensors.

The nanomaterials also exhibit excellent electrical conductivity, making them ideal candidates for high-performance electromagnetic shielding applications. Figure 13C,D shows that the conductivity exhibited by the CGO-NFC compound is capable of lighting an LED. This property enables its use in protecting against unwanted electromagnetic interference (EMI) radiation, which can disrupt the proper functioning of nearby electronic devices and also pose risks to human health [37,39].

**Figure 14:**
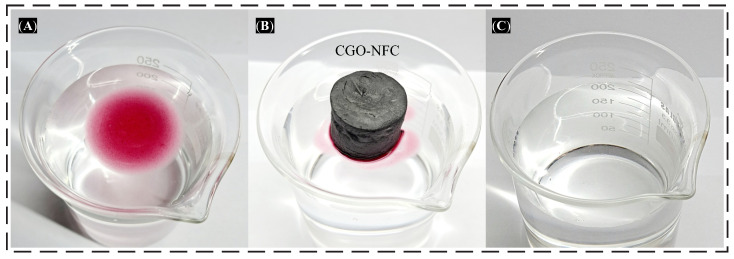
Absorption test of CGO-NFC in carmine red-dyed engine oil: (**A**) before addition of CGO-NFC, (**B**) during absorption with CGO-NFC, and (**C**) after absorption.

These GO composites synthesized with CE demonstrate excellent performance in absorbing oils and other organic compounds from aqueous solutions. It is essential for the compound to exhibit a certain degree of hydrophobicity to ensure it does not absorb water, thus ensuring the absorption of the contaminant [34,44]. To evaluate this absorption capacity, tests were conducted with the CGO-NFC nanomaterial, as illustrated in Figure 14.

It is evident that the nanomaterial demonstrated a high capacity for absorbing motor oil dyed with carmine red in just 10 min. Additionally, as observed in the figure, CGO-NFC also exhibited a significant degree of hydrophobicity when in contact with water, a crucial indicator for evaluating this type of material.

## 4. Conclusions

During the extensive investigation of the nanomaterials, adjustments were made to the synthesis and drying parameters in order to develop the best possible nanomaterial for the mentioned conditions. The challenges and complexity involved in developing high-quality nanomaterials with desired properties highlight the importance of a continuous optimization process, aiming to find the optimal combination of parameters that result in a high-quality nanomaterial while maximizing resource utilization and minimizing losses. It is a challenge that requires in-depth study and carefully planned experiments to maximize the efficiency and effectiveness of the production process of these advanced materials.

A method was proposed to compare two drying processes, resulting in a highly aligned, porous, structurally stable nanomaterial with excellent electrical, thermal, and conductive properties. FTIR spectra revealed the bonds between the GO and NFC precursors and the developed nanomaterials. Raman and XRD spectra also demonstrated the interaction between the modifying agents and the synthesized compound. Furthermore, it was observed that increasing the proportion of NFC strengthened the structure, but limited its malleability.

It is important to highlight that the parameters had a significant influence on the development of nanomaterials with the desired characteristics. The properties of the nanomaterials crosslinked with GO and NFC precursors were improved. CGO-NFC proved to be the most promising compound among the studied characteristics, in contrast to the rigidity exhibited by XGO-NFC. CGO-NFC demonstrates high potential for applications in various research and development fields.

## Figures and Tables

**Table 1:** Determination of ideal compounds.

Determination of Ideal Compounds								
Nanomaterial	GO (mg/mL)	GO:AA	GO:NFC	pH	Reduction Steps	Temperature	Time	Drying
CGO-NFC	5	1:3	1:0.25	acid	2 steps	1° step: 100 °C 2° step: 100 °C	1° step: 3 h 2° step: 6 h	lyophilization
XGO-NFC	5	1:3	1:0.25	acid	3 steps	1° step: 150 °C 2° step: 100 °C 3° step: 80 °C	1° step: 12 h 2° step: 8 h 3° step: 6 h	evaporation

**Table 2:** Physical properties of CGO-NFC and XGO-NFC.

Nanomaterial	Density g/cm${}^{3}$	Shrinkage (%)	Porosity (%)
CGO-NFC	0.0154	2.86	98.62
XGO-NFC	0.0249	6.54	98.19

**Table 3:** Vibration peaks observed by FTIR in the precursors and nanomaterials.

Wavenumber (cm^−1^)						
GO	NFC	Raw Fiber	CGO-NFC	XGO-NFC	Functional Group	Assignment
578	-	-	-	-	C-H	Methyl group bending vibration.
-	-	-	796	800	C-H	Deformation of the C-H bonds present in aromatic rings.
875	896	-	-	-	C-H	Aromatic deformation in GO and glycosidic ring vibration in amorphous domains of NFC.
1039	1026	1035	-	-	C-O	Primary alcohol stretching vibrations in GO and stretching vibrations of cellulose, lignin, and hemicellulose.
1164	1159	1155	-	-	C-O	Stretching vibrations of C-OH in GO and asymmetric stretching of ether in NFC and raw fiber.
1218	1265	1243	1298	1296	C-O	Stretching vibrations of carboxylic acid in GO and nanomaterials, and stretching of ester, ether, and phenol groups in NFC and raw fiber.
-	1317	1315	-	-	O-H	Hydroxyl group in planar bending in NFC and raw fiber.
-	1369	1369	-	-	C-H	Deformation vibration in NFC and raw fiber.
-	-	-	1402	1400	C-H3	Asymmetric deformation vibration.
-	1421–1452	1438	-	-	C-H	Methyl group deformation in NFC and raw fiber.
-	1506	1517	-	-	C=C	Stretching of the skeletal vibration of the aromatic ring in NFC and raw fiber.
1620	1594	1608	1631	1631	C=C	Stretching vibrations of the sp² carbon skeletal network in GO and stretching of the skeletal vibration of the aromatic ring in NFC, raw fiber, and nanomaterials.
1726	-	1731	-	-	C=O	Stretching vibrations of the C=O group in the carboxyl group in GO and raw fiber.
-	2889	2921	2852–2921	2852–2923	C-H	Stretching vibrations of the methyl group in NFC, raw fiber, and nanomaterials.
3213–3319	3332	3322	3467	3467	O-H	Stretching vibrations of the hydroxyl group in GO, NFC, raw fiber, and nanomaterials.

## Data Availability

Data sharing not applicable.
